# A Rare Case of Kounis Syndrome Secondary to Infliximab

**DOI:** 10.7759/cureus.44704

**Published:** 2023-09-05

**Authors:** Porfirio E Diaz-Rodriguez, Victor H Molina-Lopez, Benjamin A Gonzalez Burgos, Carlos Nieves-La Cruz

**Affiliations:** 1 Cardiology, Veterans Affairs (VA) Medical Center, San Juan, PRI; 2 Cardiology, Veteran Affairs (VA) Caribbean Healthcare System, San Juan, PRI; 3 Internal Medicine, Veterans Affairs (VA) Medical Center, San Juan, PRI; 4 Interventional Cardiology, Veteran Affairs (VA) Caribbean Healthcare System, San Juan, PRI

**Keywords:** coronary artery vasospasm, infliximab, myocardial infarction, anaphylaxis, kounis syndrome

## Abstract

Kounis syndrome (KS) is an acute coronary syndrome triggered by allergic or anaphylactic reactions. It manifests as coronary artery vasospasm, acute myocardial infarction, or coronary stent thrombosis, resulting from inflammatory cytokine release and inappropriate activation of platelets and mast cells. We present a case of an 85-year-old male with Crohn's disease who suffered anaphylaxis during infliximab infusion, culminating in non-ST myocardial infarction (NSTEMI). The patient's symptoms were effectively managed with epinephrine and diphenhydramine, and KS secondary to infliximab was diagnosed. Diagnosing KS can be challenging due to the overlapping signs of an allergic reaction and myocardial infarction. Timely recognition and appropriate management of KS are crucial to enhance patient outcomes. Therefore, healthcare providers should maintain a high index of suspicion for KS in patients with acute coronary syndromes linked to allergic reactions to optimize care and minimize potential risks. This case report underscores the significance of prompt intervention and awareness of Kounis syndrome in clinical practice.

## Introduction

Kounis syndrome (KS) is an acute coronary syndrome that occurs in the context of allergic or anaphylactic reactions, and it can manifest as coronary artery vasospasm, acute myocardial infarction, or coronary stent thrombosis [1]. First reported by Kounis and Zavras in 1991, KS is often referred to as "allergic angina" or "allergic myocardial infarction" [2]. The pathophysiological mechanism involves the release of inflammatory cytokines and inappropriate activation of platelets and mast cells, ultimately leading to myocardial ischemia [1]. Timely recognition of KS is paramount to ensure patients receive appropriate management and treatment, for improved clinical outcomes.

## Case presentation

**Figure 1:**
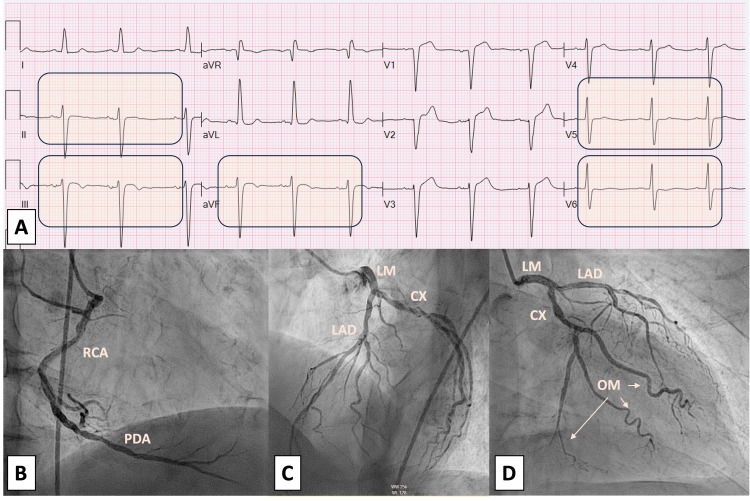
A: Electrocardiogram; B-D: Coronary angiogram Figure 1A: Electrocardiogram showing a normal sinus rhythm with left ventricular hypertrophy along with repolarization changes and nonspecific T-wave changes in lateral leads. Figure 1B: Angiography showing the right coronary artery (RCA) with non-obstructive disease. Figure 1C: Angiography showing the left main, left anterior descending, and circumflex artery with non-obstructive disease. Figure 1D: Angiography showing the left main, left anterior descending, and circumflex artery with non-obstructive disease.

We present the case of an 85-year-old male patient with a medical history of hypertension, type 2 diabetes mellitus, and Crohn's disease. The patient presented to the emergency department following a severe anaphylactic reaction that occurred a few minutes after administering his third dose of Infliximab for Crohn's disease; no premedication was given due to the fact that no prior reactions were reported. The patient exhibited notable symptoms of respiratory distress, chest pain, diaphoresis, and tachycardia, accompanied by a generalized maculopapular rash on the trunk and limbs. Vital signs at that moment were BP 190/90 mmHg, heart rate 109 bpm, O_2_ saturation 99%, and temperature 99 F. Immediate medical intervention involved the administration of intramuscular epinephrine and intravenous diphenhydramine to manage the allergic reaction. The patient’s angina was responsive to sublingual nitroglycerin. The 12-lead electrocardiogram (ECG) revealed normal sinus rhythm and left ventricular hypertrophy with repolarization changes and nonspecific T wave changes in lateral leads (Figure 1. A). Laboratory analyses demonstrated elevated high-sensitivity troponin levels at 13, 23, and 39 ng/L (reference range: 0-22 ng/L). Furthermore, the transthoracic echocardiogram (TTE) showed an ejection fraction of 40-45% with hypokinesia of the inferior wall of the left ventricle (Video 1). Consequently, urgent coronary angiography revealed non-obstructive coronary artery disease (Figure 1. B-C). The left ventriculogram revealed inferobasal hypokinesia (Video 2).

**Video 1:** Echocardiogram showing inferior wall hypokinesia

**Video 2:** Ventriculogram showing mild inferior basal hypokinesis

A comprehensive analysis of the patient's clinical presentation, which encompassed elevated cardiac enzymes, echocardiographic findings of hypokinesia, non-obstructive coronary artery disease, and manifestations of an anaphylactic reaction, led to the conclusive diagnosis of an allergic ACS triggered by Infliximab. This case underscores the significance of vigilance for potential adverse reactions in patients undergoing infliximab therapy for Crohn's disease and highlights the importance of the prompt recognition and management of such reactions to optimize patient outcomes.

## Discussion

Kounis syndrome, also known as allergic myocardial infarction or allergic angina, is a condition characterized by acute coronary events triggered by allergic or hypersensitivity reactions. Dr. Nicholas Kounis first described it in 1991 [2], and it has since gained recognition as an important clinical entity. This syndrome encompasses various allergic reactions, including drug-induced hypersensitivity, food allergies, insect bites, and anaphylaxis. There are reported cases in the literature about Kounis syndrome caused by different drugs, food, and environmental exposure [1,3] but few of them are related to infliximab [4]. The mechanism of Kounis syndrome induced by infliximab could be explained by anaphylaxis. Anaphylaxis is primarily triggered by the activation of mast cells and basophils through the cross-linking of immunoglobulin E (IgE) and FcεRI receptors. This activation leads to the rapid release of preformed mediators like histamine, tryptase, carboxypeptidase A, and proteoglycans. Subsequent activation of enzymes produces arachidonic acid metabolites, including prostaglandins, leukotrienes, and PAF. Tumor necrosis factor-alpha (TNF-α) is released as a preformed and late-phase mediator along with other cytokines and chemokines. These mediators contribute to symptoms such as vasodilation, increased vascular permeability, bronchoconstriction, and other allergic responses associated with anaphylaxis [5]. As in this case, these mediators induce coronary vasospasm, plaque erosion or rupture, platelet activation, and thrombus formation, leading to acute myocardial ischemia or infarction. While the condition is commonly seen in patients with pre-existing coronary artery disease, it can also occur in individuals with normal coronary arteries [1,2]. A review article on KS epidemiology highlighted a rapid increase in the number of identified triggers, with antibiotics (27.4%) and insect bites (23.4%) being the most common causes [6]. Patients with Kounis syndrome tend to have prolonged hospital stays (mean, 5.8±6.0 vs. 3.0±3.9 days, P<0.001) and higher rates of all-cause in-hospital death (7.0% vs. 0.4%, P<0.001) compared to patients admitted due to other allergy/hypersensitivity/anaphylactic reactions without KS, as reported by a national registry in the United States [7].

There are three primary variants of Kounis syndrome, each triggered by different factors:

Type I: This variant occurs in patients with normal coronary arteries and involves the release of inflammatory mediators during an allergic reaction, leading to temporary coronary artery spasm (vasospastic angina) without significant underlying atherosclerosis [1,8].

Type II: This variant occurs in patients with pre-existing atherosclerotic coronary artery disease. An allergic reaction can trigger the rupture of a vulnerable plaque in the coronary artery, leading to acute coronary syndrome (ACS) such as myocardial infarction (heart attack) [1,8].

Type III: This variant occurs in patients with coronary artery stents. An allergic reaction can lead to stent thrombosis, which is a potentially life-threatening complication [1,8].

The clinical manifestations of Kounis syndrome vary widely, ranging from mild angina or allergic reactions to life-threatening conditions such as myocardial infarction or cardiac arrest. Diagnosis is primarily based on the patient's clinical history, electrocardiogram (ECG) findings, and laboratory investigations [1]. ECG changes may include ST-segment elevation or depression, T-wave abnormalities, or arrhythmias. Elevated cardiac biomarkers, such as troponin, are often present, indicating myocardial injury. Management of Kounis syndrome involves identifying and treating the underlying allergic trigger alongside standard therapies for acute coronary syndromes. This may include the administration of antihistamines, corticosteroids, and vasodilators to counteract the allergic response and alleviate coronary spasms [9]. In severe cases, coronary angiography and percutaneous coronary intervention (PCI) may be necessary to restore coronary blood flow [3,9,10]. Considering Kounis syndrome in patients presenting with acute coronary syndrome and a history suggestive of an allergic reaction is crucial. However, the diagnosis can be challenging, as the symptoms of an allergic reaction and myocardial infarction can overlap [7]. Therefore, a high index of suspicion and careful evaluation are essential for an accurate diagnosis and appropriate management.

## Conclusions

This case highlights the critical significance of the early recognition and diagnosis of Kounis syndrome in patients presenting with acute coronary syndromes in the context of allergic or anaphylactic reactions. Effective management requires addressing both the underlying allergic response and providing appropriate cardiovascular care to prevent further complications and enhance patient outcomes. Healthcare providers should be vigilant in considering Kounis syndrome as a potential cause in patients experiencing acute coronary syndromes associated with allergic or anaphylactic reactions, necessitating a collaborative, multidisciplinary approach for optimal management and treatment. Raising awareness of this condition and implementing timely interventions can improve patient care and reduce the potential risks associated with Kounis syndrome.
